# Early-life nutritional and environmental determinants of thymic size in infants born in rural Bangladesh

**DOI:** 10.1111/j.1651-2227.2009.01292.x

**Published:** 2009-07

**Authors:** SE Moore, AM Prentice, Y Wagatsuma, AJC Fulford, AC Collinson, R Raqib, M Vahter, LÅ Persson, SE Arifeen

**Affiliations:** 1 MRC Keneba, MRC LaboratoriesThe Gambia; 2 MRC International Nutrition Group, Nutrition & Public Health Intervention Research Unit, London School of Hygiene & Tropical MedicineLondon, UK; 3 Department of Epidemiology, Graduate School of Comprehensive Human Sciences, University of TsukubaTsukuba, Japan; 4 Directorate of Child and Women's Health, Bramble Ward, Royal Devon and Exeter Hospital (Wonford)Exeter, UK; 5 International Centre for Diarrhoeal Disease Research, Bangladesh (ICDDR,B)Dhaka, Bangladesh; 6 Institute of Environmental Medicine, Karolinska InstitutetStockholm, Sweden; 7 International Maternal and Child Health, Department of Women's and Children's Health, Uppsala UniversitySweden

**Keywords:** Bangladesh, Birth weight, Immunity, Thymus

## Abstract

**Aim:**

The aim was to assess the impact of nutritional status and environmental exposures on infant thymic development in the rural Matlab region of Bangladesh.

**Methods:**

In a cohort of N_max_ 2094 infants born during a randomized study of combined interventions to improve maternal and infant health, thymic volume (thymic index, TI) was assessed by ultrasonography at birth and at 8, 24 and 52 weeks of age. Data on birth weight, infant anthropometry and feeding status were also collected.

**Results:**

At all ages, TI was positively associated with infant weight and strongly associated with the month of measurement. Longer duration of exclusive breastfeeding resulted in a larger TI at 52 weeks. TI at birth and at 8 weeks correlated positively with birth weight, but by 24 and 52 weeks and when adjusted for infant weight this effect was no longer present. Thymic size was not affected by pre-natal maternal supplementation or by socioeconomic status but was correlated to arsenic exposure during pregnancy.

**Conclusion:**

In this population of rural Bangladeshi infants, thymic development is influenced by both nutritional and environmental exposures early in life. The long-term functional implications of these findings warrant further investigation.

## Introduction

Early infancy is a time of rapid immunological development, where antigenic stimuli can shape the developing immune system, potentially ‘programming’ long-lasting immune responsiveness. In a developing country context where the primary focus of research and health interventions is on infectious disease control, understanding this early-life programming is critical. Recent research suggests that nutritional status in early life may impact on human immune development with, for example, a positive association observed between birth weight and antibody response to certain vaccines later in life (1–3). The precise relationship between nutritional exposures during critical periods of development and later immune function warrants further investigation.

The human thymus is a primary lymphoid organ essential for the establishment of a normal peripheral T-lymphocyte immune system. In the thymus, bone marrow-derived precursors undergo differentiation and selection, ultimately leading to the migration of positively selected thymocytes to the T-cell-dependent areas of the peripheral lymphoid organs (4,5). It has long been noted that the thymus is critically sensitive to undernutrition, with protein energy malnutrition causing atrophy of the thymus (6), highlighting the thymus as a putative target for early-life programming effects.

Post-natal development of the thymus can be assessed sonographically using a validated method to estimate a volume-related thymic index. This technique has previously been used to indicate that the thymus is sensitive to early mode of feeding, with a smaller mean thymic index observed in bottle-fed than breast-fed infants (7,8). Further, we have previously shown that an infant's thymic index tracks within individuals from birth to 12 months of age, even after adjustment for differences in body weight (9). Of note, a study from Guinea Bissau in West Africa indicated that a small thymus at birth predicts an increase in risk of infection-related mortality in infancy (10).

In rural Bangladesh, the MINIMat study has explored combinations of pre- and post-natal nutritional interventions to address the issues of maternal, fetal and infant malnutrition. In a sub-cohort of infants from the main study, we have used serial ultrasonography to explore early-life predictors of thymic development. As the concentration of arsenic in drinking water was frequently elevated in the study area, we also investigated the effects of arsenic exposure on thymic development.

## Subjects and methods

### Study population

The study was conducted in the Matlab region of Bangladesh, a rural but densely populated riverine area, situated about 60 km southeast of the capital Dhaka. The population of the Matlab region is well characterized, having participated in a demographic surveillance system (DSS) since 1963 as part of a health-related research programme led by the International Centre for Diarrhoeal Disease Research, Bangladesh (ICDDR,B). In 2001, ICDDR,B initiated the ‘Maternal and Infant Nutrition Interventions, Matlab’ (MINIMat) study, randomizing all pregnant women in Matlab to receive a combination of protein energy and micronutrient supplements. On enrolment, usually around 9 weeks of gestation, women were randomly allocated to a prenatal food supplement in combination with three separate micronutrient supplements. At the time of the study in Bangladesh, an ongoing government-supported national programme issued a protein-energy dense supplement providing 600 kcal/day to pregnant women 6 days per week. On enrolment to the MINIMat study, women were randomized to start this food supplement early in the first trimester of pregnancy (early start) or in the second trimester as the usual national programme suggests (usual start). Women were then further randomized to three micronutrient supplements: (i) the UNICEF/UNU/WHO preparation of 15 different micronutrients including 30 mg iron and 400 μg folic acid, (ii) 60 mg iron and 400 μg folic acid or (iii) 30 mg iron and 400 μg folic acid daily from week 14 during pregnancy. All supplements continued up to birth. Peri-natally, women were further randomized to receive exclusive breastfeeding counselling or the standard health counselling provided by local caregivers. The prenatal arm of the study was completed in June 2004 with a total of 3267 singleton infants born. The full protocol and main findings from the MINIMat study will be published elsewhere. For the purpose of the current study, we recruited mothers and their infants from the main MINIMat trial either at birth (for health centre deliveries) or at follow-up (for all other births).

For health centre deliveries, birth weight, length, head circumference and knee–heel lengths were measured immediately following delivery. For births occurring at home, a birth notification system was established to ensure that study staff were made aware of births as soon as they occurred. A female paramedic then visited the newborn within 72 h of birth. Birth weight was measured by electronic or beam scales, to the nearest 10 g. Recumbent length was measured using locally manufactured, collapsible length boards, precise to the nearest 1 mm.

Following delivery, infant weight and length were measured at monthly visits till 12 months of age using regularly validated standard equipment. At separate monthly visits, a female fieldworker visited each household to collect information on infant feeding practices. Full details are as described elsewhere (11). Data on feeding practices were later coded and breastfeeding status classified on the basis of current WHO recommendations (12), i.e. (i) exclusively breastfeeding (breast milk only) (ii) predominant breastfeeding (breast milk plus other liquids such as water, tea or juice) and (iii) partial breastfeeding (other food or milk in addition to breast milk). Socioeconomic status (SES) was estimated using a wealth index based on information on household assets and estimated by principal component analysis, producing a weighted score (13). Scores were grouped into quintiles.

As the study area had a high prevalence of tube wells with elevated concentrations of inorganic arsenic (As) (14), we also evaluated effects of prenatal arsenic exposure, assessed as the concentrations in maternal urine in gestational weeks 8 (early) and 30 (late), as described previously (15). The sum of metabolites of inorganic arsenic in spot urine samples was measured by hydride generation atomic absorption spectroscopy and adjusted for variation in urine dilution by specific gravity (mean 1.0012 g/mL). The means of the early and late gestation measurements were used in the current analysis.

### Thymus size assessment

Thymus size was assessed sonographically using a validated method in which the transverse diameter of the thymus and the sagittal area of its largest lobe are multiplied to give a volume-related thymic index (TI) (16). This index has been shown to correlate with thymus weight at autopsy and has been used to show that the human thymus is sensitive to environmental influences during infancy (9). Thymus size was measured using a portable ultrasound machine (Toshiba SSA 320A Justavision-200, Toshiba Medical Systems, Japan) together with a PVF-745V 5.0- to 7.0-MHz probe (Toshiba Medical Systems, UK). The TI was calculated using the mean of three measurements of both the transverse diameter and the sagittal plane. Measurements were performed in triplicate by trained health practitioners.

TI was assessed at four time points during infancy: within 24 h of birth (health centre deliveries only) and at 8, 24 and 52 weeks of age. Infants were scanned in the supine position, using a transternal approach; sonography was not performed while the infant was crying. Owing to a delay with the start of TI assessment, a large proportion of infants had passed 16 weeks by the time the study started; this resulted in more infants with TI measured at the latter two time points.

### Ethics

The main MINIMat study and the thymic size addendum were approved by the Research Review and Ethical Review Committees, ICDDR,B, Dhaka, Bangladesh. The thymic size addendum was additionally approved by the Ethics Committee at the London School of Hygiene and Tropical Medicine. Written informed consent was obtained from all participating mothers following an explanation of the study in their own language.

### Statistical analysis

Comparisons among group means were made using two-sample *t*-tests. Associations between TI at each time point were assessed by correlation analysis, with TI measurements adjusted for season, weight and sex of the infant. TI was regressed on these covariates, separately at each time point, and the correlations between time points were calculated for the resulting residual (difference between the observed and predicted values). The effect of seasonality on TI size was assessed by fitting a truncated Fourier series regressed against TI (17). To test whether seasonality changed with age, a pooled analysis of all time points was performed using a multi-level model to allow for the correlation between measurements at different time points on the same child. To simplify the analysis, only the interaction between time point and the first-order sine and cosine terms [sin(θ_i_) and cos(θ_i_)] was considered. The effect of birth size on TI at each time point was assessed by multiple regression analysis, controlling for sex, infant length and season of measurement. Infant length was included in the models since thymic size is expected to be related to infant size. Thus at birth, the relationship between TI and birth weight should be interpreted in terms of weight-for-length. Since infants’ lengths tend to track along their starting centile, current size will be strongly correlated with birth length, and the TI–birth weight relationship will also have a similar interpretation at subsequent time points. For seasonality, the first two pairs of Fourier terms were fitted, i.e. the terms in β_3_ to β_6_, in the following model (17):
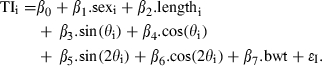
(1) In order to investigate the effect of arsenic [As] on TI, a random effects model was fitted with As as a quadratic, while controlling for sex and infant length, age and season of measurement and pooling data from all time points (i.e. adding the terms {[As] and [As]− mean([As])^2^} to the model shown above). SES fitted as a five-level categorical variable was added to the model in order to check for confounding with [As].

In the regression analyses, models were compared using the ANOVA *F*-test and the likelihood ratio test for ordinary multiple regression and multi-level models, respectively.

*Z*-scores were calculated using the Child Growth Standards of the WHO, using the WHO Anthro 2005 software and macros (http://www.who.int/childgrowth/software/en/). All statistical analyses were performed using DataDesk, version 6 for Windows from Data Description Inc. (Ithaca, NY, USA) or Stata 9 from Stata Corp. (College Station, TX, USA).

## Results

Anthropometric characteristics of the infants at each of the time points when TI was assessed are detailed in Table 1. Mean (range) maternal weight and BMI at 30-week gestation were 50.7 kg (33.6–95.8) and 20.2 kg/m^2^ (18.3–31.2), respectively, and a strong positive association was observed between both measures and infant weight at birth (p ≤ 0.0001 for both). A total of 1168 infants had their TI measured within 24 h of birth. When compared with infants who did not have TI measured at birth, infants who had TI measured at birth were significantly heavier (2747 g vs. 2673 g; p < 0.0001) and had a significantly longer gestational age (39.05 weeks vs. 38.76 weeks; p = 0.0004). No differences were observed with birth length (47.7 cm vs. 47.8 cm; p = 0.1821).

**Table 1:** Infant characteristics at birth and at 8, 24 and 52 weeks of age

		Males		Females	
	N	Mean	SD	Mean	SD
Birth					
Weight (kg)	1168	2.79	0.39	2.70	0.38
Length (cm)	1168	48.06	2.01	47.31	2.01
Gestational age (week)	1148	38.97	2.13	39.13	2.06
Week 8					
Weight (kg)	1650	4.77	0.62	4.41	0.57
Length (cm)	1637	55.7	2.19	54.5	2.13
WHZ	1635	−0.05	1.25	−0.09	1.07
Week 24					
Weight (kg)	1662	7.02	0.87	6.45	0.81
Length (cm)	1655	64.5	2.30	62.7	2.21
WHZ	1652	−0.28	1.15	−0.21	0.98
Week 52					
Weight (kg)	2881	8.30	1.08	7.66	1.00
Length (cm)	2860	71.7	2.59	69.9	2.53
WHZ	2860	−0.76	1.11	−0.73	1.05

Data are for infants for whom a measure of thymic index plus the relevant anthropometric measure is available at each time point.

WHZ = weight-for-height standard deviation score using WHO reference data.

Mean (95% CI) TI at birth was 27.6 (27.1–28.0) and was positively correlated to birth weight and birth length (weight r = 0.310, length r = 0.242; p ≤ 0.0001 for both). A significant but weak positive correlation was observed between TI at birth and both maternal weight and BMI at 30-week gestation (r = 0.088, p = 0.0026 and r = 0.087, p = 0.0029, respectively) but not for parity (p = 0.5161). However, all other associations became non-significant once adjusted for infant birth weight.

**Figure 1:**
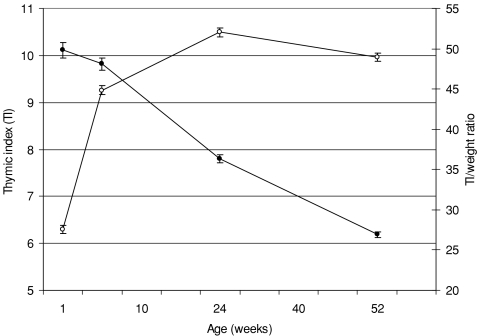
Mean thymic index (open circles) and thymic index expressed in relation to body weight (closed circles) by age. Data are expressed as means and 95% CIs.

As a consequence of the delay in starting these measurements, the number of infants with TI assessed at each time point during infancy increased from 1715 and 1704 at 8 and 24 weeks, respectively, to 2094 infants at 52 weeks. Mean TI increased rapidly from birth to week 8, reaching maximum size at week 24 and then declining to the week 52 measurement (Fig. 1). TI was strongly positively correlated to current infant weight and length at all time points (p ≤ 0.0001 for all). Also illustrated in Figure 1 is the change in TI expressed in relation to body weight; relative to infant weight, TI was largest at birth and smallest at 52 week. TI was significantly related to sex at all age points, with a larger thymus observed in male infants, although following adjustment for infant weight, these differences only remained significant for the measurements taken at 24 and 52 weeks (24 week, 52.9 vs. 51.2, p = 0.007; 52 week, 50.1 vs. 47.9, p ≤ 0.0001).

**Figure 2:**
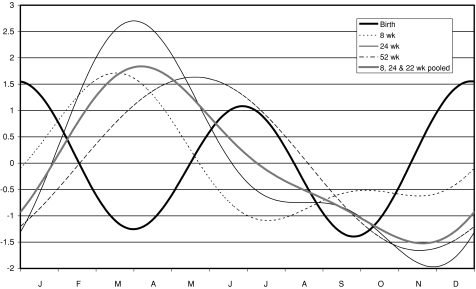
Relative size of thymus variation with time of year plotted at birth and at 8, 24 and 52 weeks of age. The curves are the seasonal component of equation (1) fitted by multiple regression. They show the mean TI difference from the overall average at different times of the year. Post-natally, the thymus is largest in the first half of the year and smallest in the second half of the year. The picture is rather different at birth where there appeared to be two peaks: at the turn of the year and at mid-year. The difference between the patterns at the different post-natal ages is not significant but these do differ significantly from the pattern at birth.

The effect of month of measurement on mean TI at each time point and for all time points pooled is illustrated in Figure 2. Adjusted for sex and infant length at the time of measurement, a significant association with season of measurement was observed at all time points, with the exception of the measurements made at 8 weeks of age: Birth p = 0.0012; 8-week p = 0.0739; 24-week p ≤ 0.0001 and 52-week p = 0.0002. There are three broad seasons in Bangladesh: the hot and dry season (March to June), the monsoon season (July to September) and winter (October to February). At birth, mean TI showed a bimodal pattern, with largest TIs observed when measured in June and December and smallest in March and September. For the measurements made when the infants were 8, 24 and 52 weeks old, TIs peaked only once during the hot and dry months of the year falling in size during the monsoon and winter months. When the measurements at all time points were pooled, the seasonal pattern differed significantly between time points (likelihood ratio test χ^2^ on 6 df = 21.18; p = 0.0017). This difference was almost entirely due to the difference between the pattern at birth and the patterns observed post-natally (likelihood ratio test χ^2^ on 2 df = 12.51; p = 0.0019) while no further difference was detected between the post-natal patterns (likelihood ratio test χ^2^ on 4 df = 8.67; p = 0.070). The seasonal pattern with the time points pooled is also illustrated in Figure 2.

Using an intention-to-treat analysis, we also explored the relationship between pre-natal supplementation and exclusive breastfeeding counselling and post-natal thymic development. No associations were observed between any of the prenatal interventions (food or micronutrients) and TI at any time point (data not presented). According to lactation counselling group, no associations were observed between TI at birth and at 24 or 52 weeks, but at 8 weeks, infants born to mothers who had received counselling in exclusive breastfeeding had a marginally significantly smaller TI than those who had not (44.3 vs. 45.5; p = 0.0415). Using mother-reported duration of exclusive breastfeeding (assessed in blocks of 15 days) as the exposure variable, no associations were observed with the measures of TI taken at 8 and 24 weeks. However, at 52 weeks, infants who had been exclusively breastfed for greater than the median duration of days of exclusive breastfeeding (59.9 days) had a significantly larger thymus than those who had not (<59.9-day TI = 48.3, >59.9-day TI = 49.7; p = 0.0070). This remained the case when adjusted for infant length and sex (p = 0.0129).

The median As concentration in maternal urine for the 1556 mother–infant pairs who had both As data and TI measured at week 52 was 102 μg/L (range 5.5–1150 μg/L). The analysis of the effect of prenatal As exposure on TI revealed a complex relationship. The simple linear trend alone was not significant (p = 0.74) but the addition of the quadratic term gave a highly significant fit (χ^2^ = 12.93 on 2 df; p = 0.0016): TI was negatively associated with As, but this association diminished at high-exposure levels. Overall, however, As exposure explained only 4% of the variance in TI. Since the diminishing effect of As was unexpected, we considered the possibility that it might have resulted from confounding with SES. SES did correlate strongly with both TI and As, although the association with TI was entirely explained by its association with infant size. When added to the regression model, already controlled for infant length, SES had a negligible effect on the relationship between As and TI.

Table 2 shows the correlation between TI at each age point, adjusted for size and sex of the infant and for the season of measurement. TI at each age was significantly correlated with the infant's previous TI, suggesting that an infant's thymic size ‘tracks’ within individuals. We then looked at the influence of birth weight on thymic development in infancy. Table 3 illustrates the output from multiple regression analysis of birth weight on thymic size, adjusted for sex, infant length and season of measurement, at each time point. Birth weight was significantly related to TI at birth and at 8 weeks, but by 24 and 52 weeks, the association became non-significant.

**Table 3:** Association between birth weight and thymic index at different time points

Time point	β-Coefficient	SE	p-value	95% CI
Birth	5.996	0.856	≤0.0001	4.316, 7.676
Week 8	2.840	1.010	0.005	0.859, 4.821
Week 24	0.579	0.841	0.491	−1.071, 2.228
Week 52	0.556	0.681	0.415	−0.781, 1.892

Data are derived from regression analysis adjusting for sex, season and infant length at the time of measurement. The β-coefficient gives the increase in the thymic index at different ages associated with each kg increase in birthweight. This association clearly fades rapidly in the first year of life.

**Table 2:** Correlation matrix for adjusted thymic index (adjusted for season, sex and infant size)

	Birth	Week 8	Week 24	Week 52
Birth	1.000			
Week 8	0.340	1.000		
	*(0.282, 0.396)*			
	*965*			
Week 24	0.152	0.284	1.000	
	*(0.081, 0.221)*	*(0.226, 0.339)*		
	*777*	*1074*		
Week 52	0.177	0.216	0.207	1.000
	*(0.100, 0.251)*	*(0.155, 0.276)*	*(0.149, 0.264)*	
	*660*	*987*	*1107*	

Data tabulated are correlation coefficients, their 95% confidence intervals (based on Fisher's transformation) and sample size at each time point. All correlations are significant at the p < 0.0001 level.

## Discussion

The relationship between nutritional status and human immune function is complex, reflected in the wide range of both nutritional exposures and immune markers assessed in nutritional immunology research. It is however well reported that a deficiency of both macro- and micronutrients can compromise immunity, and recent research now links nutritional exposures early in life with alterations in functional immunity that persist beyond childhood. In the current study, we used sonographic assessment of thymic size to assess immune development in a large cohort of Bangladeshi infants born during a trial of prenatal multiple micronutrients and early food supplementation. We have shown that thymic development during the first year of life is sensitive to a number of nutritional and environmental exposures including birth weight, season of measurement, duration of exclusive breastfeeding and maternal exposure to environmental arsenic. All of these effects are independent of the expected allometric scaling of organ size.

The primary role of the human thymus is the site for the differentiation of bone-marrow-derived T-cell precursors into positively selected thymocytes. This process involves sequential expression of various proteins and rearrangements of T-cell receptor genes (5). During stress, such as that induced by severe malnutrition, the thymus undergoes a severe atrophy due to apoptosis-induced depletion, particularly affecting the immature CD4+ and CD8+ cells, as well as a decrease in cell proliferation (4). This atrophy can be detected by sonographic assessment (18) and, when induced by malnutrition, can be reversed with nutritional rehabilitation (6).

In the current study, thymic size was assessed within 24 h of delivery for sub-centre deliveries, and at 8, 24 and 52 weeks in all other infants. As has been observed elsewhere (9,19,20), mean TI increased rapidly from birth, reached maximal size by 24 weeks, and then declined to the measurement made when the infants were 52 weeks old. When expressed in relation to body weight, TI was greatest at birth, falling to the measurement taken at 52 weeks.

A strong effect of month of measurement was observed at all time points, although this did not reach statistical significance when the infants were aged 8 weeks. A strong effect of the season of measurement was also observed in The Gambia, where thymic size was consistently smaller when measured in the annual wet season (9). The seasonal patterns observed in the current study from Bangladesh are a little less clear, with a distinct difference observed between the pattern shown at birth and that at all other time points. At birth, TI peaked twice during the year: once in the cool winter months and once in the hot summer months. For all other time points however, a single peak in the hot dry season was observed, falling rapidly during the annual monsoon season. We have previously shown that, in countries with a strong seasonal variation in a number of environmental exposures, antibody responses to vaccination are influenced by month of vaccination (21). Although the precise aetiology of these seasonal effects on TI in the current study, and particularly the apparent difference between birth and all subsequent measures, cannot be elucidated from the data available, we speculate that it is the result of a co-stimulatory effect of seasonally variable environmental antigens. The long-term impact of this seasonal variation also warrants further investigation.

In rural Gambia, the decrease in the thymic size during the annual wet season corresponded with a decrease in levels of breast milk interleukin (IL)-7 levels (22), suggesting a putative role of breast milk immune proteins in thymic development. In the current study, infants exclusively breastfed for a longer duration had a larger thymic index at 52 weeks. A number of previous studies have highlighted a beneficial role of breastfeeding in thymic development (7,8). In populations such as Matlab, where infectious disease morbidity and mortality during infancy remains high, this finding could indicate an important beneficial role of exclusive breastfeeding in immune development in the infant beyond the period of exclusive breastfeeding itself.

The region of Matlab represents an area of Bangladesh with a high prevalence of arsenic-contaminated tube well water (14). Chronic exposure to inorganic arsenic is associated with various forms of cancer, skin effects, diabetes, hypertension, liver and neurotoxicity, and limited studies indicate effects on the immune system (23,24). Arsenic is known to pass through the placenta to the fetus (25) and we have observed a negative association between maternal arsenic exposure in pregnancy and birth weight in the present cohort (26). Similarly, our recent study in the same area showed that exposure to arsenic via drinking water during pregnancy significantly increased the risk of fetal loss and, in particular, infant mortality (27).

The latter would be compatible with a prenatal effect on immune function and we are currently investigating child mortality in relation to prenatal arsenic exposure in more depth. Although we have recently shown that breastfeeding protects against early-life exposure to arsenic (28), non-exclusively breast-fed infants will be exposed to arsenic through water and weaning foods prepared using contaminated water and this exposure will obviously increase with age. In the current study, a curvilinear relationship was observed between maternal exposure to arsenic and infant thymic size with a lower TI in infants born to mothers with higher As exposure suggesting that prenatal As exposure negatively impacts on the development of the infant thymus. The reason for the stronger association in the lower exposure interval is not known, but is in accordance with our other studies on birth weight and infant mortality (26,27). Further research is needed to investigate the dose–effect relationship, the most critical windows of exposure, as well as the importance of the effects of early-life exposure to environmental As on thymic development and on later immune competence in infants.

The main MINIMat study assessed the effects of prenatal multiple micronutrients and early food supplements on birth outcomes. Using an intention-to-treat analysis, we found no effect of prenatal supplementation on thymic size, a finding perhaps not surprising given the lack of effect of supplementation on birth weight (unpublished data). We did however observe a positive correlation between birth weight and thymic size, an effect that persisted until the infants were 8 weeks of age, irrespective of the current infant size. This finding suggests an *in utero* influence on thymic development, possibly mediated by maternal nutritional status. Recent research has focused on the hypothesis that nutritional status during fetal life and early infancy may be critical for immune development, with long-lasting effects on functional immunity (29). In Filipino adolescents (1) and Pakistani adults (2,3), for example, a weaker antibody response to vaccination with polysaccharide antigens was observed in subjects born with a lower birth weight. Together with the findings from the current study, these observations highlight the need for further randomized control intervention studies assessing the impact of early-life nutrition on infant immune development in populations with a marginal nutritional status.

A limitation of the current study is the reliance on thymic size as an indicator for early immune development with no direct assessment of thymic function included. Little published data exist to correlate thymic size to function during infancy. In a small study of Danish infants, a positive correlation was observed between thymus size and the proportion of CD8+ cells in peripheral blood and the CD4/CD8 ratio at 10 months of age (30). In a sub-cohort of 310 infants from the current study, thymic size was shown to correlate positively with signal-joint T-cell receptor rearrangement circles (TRECs) (Raqib et al., unpublished observations). TRECs are formed during the generation and expression of the T-cell antigen receptor (31) and levels in peripheral blood T-cells are increasingly used as a direct measure of thymic output. The positive correlation observed between thymic size and TREC levels in these infants provides important direct evidence that thymic size is a measure of function in this cohort of Bangladeshi infants. Where possible, further studies of thymic size development should incorporate additional measures of function.

In conclusion, we have shown that the human thymus is sensitive to a number of nutritional and environmental exposures and that these effects appear to persist throughout early infancy. The long-term consequence of having a smaller thymus are yet to be fully understood, although in a study from Guinea-Bissau, West Africa, a smaller TI at birth was associated with increased infant mortality (10) suggesting a predictive role of the thymus in survival from infectious diseases in early infancy. We are currently following up the MINIMat cohort of infants to assess immune function now that the children are 4.5 years of age; analysis within this cohort by early-life thymic size will help understand the relationship between early-life nutritional and environmental exposures and long-term immune function.
